# Comparison of Clinical Features and Outcomes between SARS-CoV-2 and Non-SARS-CoV-2 Respiratory Viruses Associated Acute Respiratory Distress Syndrome: Retrospective Analysis

**DOI:** 10.3390/jcm11082246

**Published:** 2022-04-17

**Authors:** Manbong Heo, Jong Hwan Jeong, Sunmi Ju, Seung Jun Lee, Yi Yeong Jeong, Jong Deog Lee, Jung-Wan Yoo

**Affiliations:** 1 Department of Internal Medicine, Gyeongsang National University Hospital, Jinju 52727, Korea; cough@kakao.com (M.H.); bechem6939@naver.com (J.H.J.); smangel518@naver.com (S.J.); juny2278@naver.com (S.J.L.); dr202202@naver.com (Y.Y.J.); ljd8611@nate.com (J.D.L.); 2 School of Medicine, Gyeongsang National University, Jinju 52828, Korea

**Keywords:** SARS-CoV-2, non-SARS-CoV-2 respiratory viruses, acute respiratory distress syndrome, mortality

## Abstract

Although a few studies comparing severe acute respiratory syndrome coronavirus 2 (SARS-CoV-2) and non-SARS-CoV-2 respiratory viruses have been reported, clinical features and outcomes comparing SARS-CoV-2 and non-SARS-CoV-2 respiratory viruses associated acute respiratory distress syndrome (ARDS) are still lacking. We retrospectively identified patients with SARS-CoV-2 (November 2020 to January 2022) and non-SARS-CoV-2 respiratory viruses associated ARDS (February 2015 to November 2020) at a single tertiary hospital. Their clinical data were obtained by medical record review. All viral infections were confirmed by RT-PCR. Thirty-one SARS-CoV-2 and seventy-one patients with non-SARS-CoV-2 respiratory viruses associated ARDS patients were identified. Influenza (62%) was the most common in non-SARS-CoV-2 respiratory viruses associated ARDS patients. Patients with SARS-CoV-2 were more likely to be female and had higher body mass index, lower clinical frailty, APACHE II, and SOFA score than those with non-SARS-CoV-2 respiratory viruses. All patients with SARS-CoV-2 were treated with corticosteroids and used more high-flow nasal oxygen than those with non-SARS-CoV-2 respiratory viruses. The concomitant respiratory bacterial infection was significantly higher in non-SARS-CoV-2 respiratory viruses than SARS-CoV-2. Although there were no significant differences in the 28-, 60-day, and in-hospital mortality rates between SARS-CoV-2 and non-SARS-CoV-2 respiratory viruses associated ARDS, the duration of mechanical ventilation and length of hospital stay were significantly longer in patients with SARS-CoV-2 than those with non-SARS-CoV-2 respiratory viruses. Although the severity of illness and the concomitant bacterial infection rate were lower in patients with SARS-CoV-2 associated ARDS, mortality rates did not differ from non-SARS-CoV-2 respiratory viruses associated ARDS.

## 1. Introduction 

Acute respiratory distress syndrome (ARDS) is the major critical condition requiring hospitalization in the intensive care unit (ICU) and invasive respiratory support, including mechanical ventilation or extracorporeal membrane oxygenation (ECMO) [1,2]. Despite advances in caring for critically ill patients, ARDS still results in high mortality rates [3,4].

The coronavirus disease 19 (COVID-19) pandemic, caused by the severe acute respiratory syndrome coronavirus 2 (SARS-CoV-2), is still ongoing and burdens unprecedented healthcare systems worldwide. The clinical course is a wide range from asymptomatic to life-threatening conditions [5,6]. Pneumonia is the most common lower respiratory presentation from COVID-19 [7,8]. Severe COVID-19 pneumonia frequently developed and progressed to acute respiratory distress syndrome (ARDS) requiring intensive care and several studies have reported worse outcomes in critically ill patients with COVID-19 were high [9,10,11,12,13,14]. Before the emergence of the COVID-19 pandemic, non-SARS-CoV-2 respiratory viruses infection was not uncommon in critically ill patients admitted to the ICU, and such patients often received invasive mechanical ventilation [15]. Non-SARS-CoV-2 respiratory viruses also cause ARDS and result in a high mortality rate in the ICU [16]. Therefore, the burden of non-SARS-CoV-2 respiratory viruses associated ARDS has not been ignored [17]. Several studies have compared the clinical characteristics and outcomes of critically ill patients with SARS-CoV-2 and non-SARS-CoV-2 respiratory viruses, especially influenza [18,19,20]. A few studies have included non-influenza respiratory viruses, [21,22] which also presented in severe pneumonia progressing to ARDS in critically ill patients [23,24]. However, more clinical studies are needed to clarify the features between SARS-CoV-2 associated ARDS and non-SARS-CoV-2 respiratory viruses associated ARDS. 

This study aimed to evaluate and compare the clinical characteristics and outcomes of patients with SARS-CoV-2 and non-SARS-CoV-2 respiratory viruses associated ARDS. 

## 2. Materials and Methods

### 2.1. Patients 

Between November 2020 and January 2022, medical records of patients aged ≥18 years with SARS-CoV-2 associated ARDS requiring invasive mechanical ventilation were reviewed and their clinical data were analyzed, retrospectively. SARS CoV-2 infection was confirmed by a positive result on a reverse transcriptase-polymerase chain reaction (RT-PCR) assay using specimens including nasopharyngeal swabs, sputum, or endotracheal aspirates. In cases of non-SARS-CoV-2 respiratory viruses associated ARDS, between February 2015 and November 2020, the medical records of patients aged ≥18 years who were hospitalized in a medical ICU and received invasive mechanical ventilation were searched and their clinical data were analyzed. We excluded clinical data analysis of patients aged under 18 years old or who were under the status of do-not-intubate. 

### 2.2. Data Collection

Respiratory viral infection was confirmed by RT-PCR using respiratory specimens, such as bronchial and endotracheal aspirates, nasopharyngeal swabs, or sputum. The AdvanSure^TM^ RV RT-PCR (LG Chemistry, Seoul, Korea) assay based on the multiplex PCR method was used to identify respiratory viruses (influenza virus, parainfluenza virus, respiratory syncytial virus, adenovirus, metapneumovirus, rhinovirus, bocavirus, and coronavirus). Patients with rhinovirus or non-SARS-CoV-2 coronavirus were excluded from this analysis. Both SARS-CoV-2 and non-SARS-CoV-2 respiratory viruses associated ARDS fulfilled the Berlin diagnostic criteria [25]. Baseline characteristics, the severity of illness (Acute Physiology and Chronic Health Evaluation II, APACHE II), organ dysfunction (sequential Organ Failure Assessment, (SOFA)), clinical features, and management (drugs, respiratory support, etc.) were assessed at the initiation of invasive mechanical ventilation. Additionally, laboratory and ventilator parameters, microbiologic data (types of respiratory viruses, co-respiratory bacterial infection, etc.) at the initiation of invasive mechanical ventilation, and clinical outcomes (duration of mechanical ventilation, ventilator liberation, length of hospital stay, and mortality) were analyzed. 

### 2.3. Study Outcomes

The clinical outcomes for this study included 28-, 60-day, and in-hospital mortality rates, duration of mechanical ventilation, length of hospital stay, the proportion of ventilator liberation, and tracheostomy. 

### 2.4. Statistical Analysis

Categorical variables are expressed as numbers and percentages and were analyzed using the chi-square or Fisher’s exact test. Non-categorical variables are expressed as medians and interquartile ranges (IQRs) and were compared using the Mann–Whitney U test. Log-rank tests were used to compare 60-day mortality and are depicted using the Kaplan-Meier method. All data were analyzed using the SPSS software version 22.0 (IBM Corp., Armonk, NY, USA). Figures were generated using Prism 5.01 (GraphPad Software Inc., San Diego, CA, USA).

## 3. Results

### 3.1. Comparison of Characteristics between SARS-CoV-2 and Non-SARS-CoV-2 Respiratory Viruses Associated ARDS

During the study period, 102 patients were identified (31 with SARS-CoV-2 and 71 with non-SARS-CoV-2 respiratory viruses associated ARDS). Clinical features are compared and presented in Table 1. The median patient age was 69.5 years. The proportion of male patients with SARS-CoV-2 associated ARDS was significantly lower than that of non-SARS-CoV-2 respiratory viruses associated ARDS (45.2% vs. 70.4%, *p* = 0.025). Patients with SARS-CoV-2 associated ARDS had a lower clinical frailty score than those with non-SARS-CoV-2 respiratory viruses associated ARDS (2 vs. 3, *p* < 0.001). The median APACHE II and SOFA scores were significantly lower in patients with SARS-CoV-2 associated ARDS than in those with non-SARS-CoV-2 respiratory viruses associated ARDS. Shock was observed less frequently in patients with SARS-CoV-2 associated ARDS. During high-flow nasal cannula oxygen therapy, remdesivir was administrated to 77.4% (24/31) of patients with SARS-CoV-2 associated with ARDS. Neuraminidase inhibitors, such as oseltamivir or peramivir, were administered to all 44 patients with influenza-associated ARDS. In the case of other respiratory viruses, antiviral agents were not used. All patients with SARS-CoV-2 associated ARDS received corticosteroids, whereas 30% of subjects with non-SARS-CoV-2 respiratory viruses associated ARDS received corticosteroids. The use of high-flow nasal oxygen therapy was significantly higher in patients with SARS-CoV-2 associated ARDS than in those with non-SARS-CoV-2 respiratory viruses associated ARDS (87.1% vs. 47.9%, *p* < 0.001). 

### 3.2. Comparison of Laboratory, Ventilator Parameters, and Microbiologic Results

Laboratory and, ventilator parameters and microbiologic results are shown in Table 2. C-reactive protein and procalcitonin levels were significantly lower in patients with SARS-CoV-2 associated ARDS than in those with non-SARS-CoV-2 respiratory viruses associated ARDS. Albumin, lactate, and NT-pro-BNP levels were also significantly lower in patients with SARS-CoV-2 associated ARDS. The median PaO_2_/FiO_2_ reflecting oxygenation status tended to be lower in patients with SARS-CoV-2 associated ARDS than in those with non-SARS-CoV-2 associated ARDS (87 mmHg vs. 105 mmHg, *p* = 0.099). The positive end-expiratory pressure on invasive mechanical ventilation was significantly higher in patients with SARS-CoV-2 associated ARDS than in those with non-SARS-CoV-2 respiratory viruses associated ARDS (10 cmH_2_O vs. 8 cmH_2_O, *p* = 0.007). In terms of types of respiratory viruses in patients with non-SARS-CoV-2 associated ARDS, the proportion of influenza virus was 62%, followed by metapneumovirus (11.3%). There were significantly fewer co-respiratory bacterial infections in patients with SARS-CoV-2 than those with non-SARS-CoV-2 respiratory viruses (50.7% vs. 9.6%, *p* < 0.001). In patients with non-SARS-CoV-2 respiratory viruses associated ARDS, *Staphylococcus aureus* was the most common co-respiratory bacteria.

### 3.3. Comparison of Clinical Outcomes

The clinical outcomes are presented in Table 3. The duration of mechanical ventilation was significantly longer in patients with SARS-CoV-2 associated ARDS than in those with non-SARS-CoV-2 respiratory viruses associated ARDS (18 days vs. 8 days, *p* < 0.001). The proportion of ventilator liberation did not differ between the groups. The median length of hospital stay was significantly longer in patients with SARS-CoV-2 associated ARDS than in those with non-SARS-CoV-2 respiratory viruses associated ARDS. (28 days vs. 14 days, *p* = 0.001). Overall in-hospital mortality was 53.9%. There were no differences in the 28- and 60-day mortality rates between the two groups. 

## 4. Discussion

The current study compared the characteristics and outcomes of SARS-CoV-2 and non-SARS-CoV-2 respiratory viruses associated ARDS. The findings of the study showed that compared with features of patients with non-SARS-CoV-2 respiratory viruses associated ARDS, patients with SARS-CoV-2 initially exhibited lower clinical frailty less severity of illness, and organ dysfunction. However, their clinical outcomes were similar to those of patients with non-SARS-CoV-2 respiratory viruses, and the duration of invasive mechanical ventilation and hospitalization was longer. 

ARDS is a common and devastating clinical condition, which contributes to high mortality in the ICU. Pneumonia is a common risk factor to cause it [1,2]. Respiratory virus is not an uncommon pathogen in pneumonia [26]. Respiratory viral infections are commonly identified in critically ill patients with severe pneumonia admitted to the ICU [15]. These conditions frequently develop and progress to ARDS [16]. In the previous H1N1 influenza pandemic, for example, influenza-induced ARDS was substantially attributed to high mortality in critically ill patients [27,28,29].

COVID-19, which is caused by SARS-CoV-2 infection, shows diverse clinical presentations from asymptomatic to severe cases, especially ARDS [5,6,7]. Several studies reported a high mortality rate in critically ill patients with SARS-CoV-2 associated acute respiratory failure or ARDS receiving invasive mechanical ventilation [9,10,11,12,13]. Lim et al., in their meta-analysis, reported that the estimated case fatality rate for patients with SARS-CoV-2 receiving invasive mechanical ventilation was 45% [14]. The host immune response to SARS-CoV-2 infection elicits pulmonary inflammation by releasing various pro-inflammatory cytokines and recruiting inflammatory cells, which results in diffuse alveolar damage and severe hypoxemia [7,8,30].

Considering the shared pathogenesis of respiratory tract infection and health burden in critically ill patients between SAR-CoV-2 and non-SARS-CoV-2 respiratory viruses, clarifying the clinical characteristics and outcomes between the two groups in critically ill patients has gained attention. Several studies have compared characteristics and outcomes of SARS-CoV-2 and influenza viral infection in critically ill patients [18,19,20]. Tang et al. compared the clinical characteristics and outcomes between SARS-CoV-2 and H1N1 ARDS [18]. They showed lower severity of illness and organ dysfunction scores in SARS-CoV-2 associated ARDS than those with H1N1 ARDS, but there was no significant difference in in-hospital mortality between the two groups. Cobb et al. reported that the hospital mortality rate was significantly higher in 65 critically ill patients with SARS-CoV-2 than in 74 with influenza (40% vs. 19%, *p* = 0.006) [19]. In a study including non-influenza respiratory viruses as a comparator, Richard-Belle et al., reported that critical care patients with SARS-CoV-2 had higher acute hospital mortality rates than those with other viral pneumonia (42% vs. 24%). Recently, Hedberg et al. reveal in their retrospective study that SARS-CoV-2 was associated with increased ICU admission and higher 30-day mortality in an ICU-admitted adult cohort (26% for SARS-CoV-2, 19% for influenza, 25% for respiratory syncytial virus, and 14% for other viruses) [22].

In terms of the clinical significance of non-influenza viral infection in critically ill patients, we reviewed cases of non-influenza respiratory viruses associated ARDS and categorized them as non-SARS-CoV-2 respiratory viruses. Influenza viruses were the most common non-COVID-19 respiratory viruses. In our study, patients with SARS-CoV-2 associated ARDS had several characteristics distinct from those with non-SARS-CoV-2 respiratory viruses associated ARDS. BMIs were higher in SARS-CoV-2 associated ARDS. Patients with SARS-CoV-2 associated ARDS had significantly lower clinical frailty scores, APACHE II, and SOFA scores at the time of invasive mechanical ventilation, which is consistent with previous studies [18,19]. In contrast to the lower trend of PaO_2_/FiO_2_ in subjects with SARS-CoV-2 associated with ARDS, levels of CRP and procalcitonin were significantly higher in those with non-SARS-CoV-2 respiratory viruses associated with ARDS. A significantly higher elevation in inflammatory markers may be attributed to a high proportion of early co-respiratory bacterial infections in patients with non-SARS-CoV-2 respiratory viruses associated with ARDS. 

To capitalize on the survival benefit of corticosteroids, such as low-dose dexamethasone in critically ill patients with SARS-CoV-2 receiving invasive mechanical ventilation [31], we noted that all patients with SARS-CoV-2 received corticosteroids. However, due to the controversy regarding the role of corticosteroids in ARDS and the harmful effects of corticosteroids in severe influenza pneumonia, there was less use of it in non-SARS-CoV-2 respiratory viruses associated ARDS [32,33,34]. In our hospital, unfortunately, the prone position in patients with SARS-CoV-2 associated ARDS could not be performed because of the inexperience of working staff in the study period.

The duration of mechanical ventilation and hospitalization was significantly longer in patients with SARS-CoV-2 associated ARDS than in non-SARS-CoV-2 respiratory viruses associated ARDS. This finding is in line with previous reports [19,20]. Possible explanations for a longer duration of mechanical ventilation and hospital stay in patients with SARS-CoV-2 associated ARDS are that recovery of the injured lung from SARS-CoV-2 may be slower than a non-SARS-CoV-2 respiratory virus and all use of corticosteroid for SARS-CoV-2 associated ARDS is a well-known risk factor for super-infection; for example, pulmonary aspergillosis in frequently co-infected during the management of critically ill patients with SAR-CoV-2 infection. To maintain invasive respiratory support, there was a higher rate of tracheostomy trend toward SARS-CoV-2 associated ARDS. Both groups had high mortality rates in 28-day, 60-day and in-hospital, but there were no significant differences. Although lower severity of illness and organ dysfunction were presented in SARS-CoV-2 associated ARDS, progression of lung injury during invasive mechanical ventilation or lately ventilator-associated lower respiratory bacterial and fungal super-infection such as *aspergillus* may be associated with high mortality lately [35,36,37,38].

The present study had several limitations. First, bias cannot be avoided in a retrospective study including a small size number of patients in a single center. Second, most data on non-SARS-CoV-2 respiratory viruses were analyzed before the emergence of the COVID-19 pandemic, and historical comparisons were inevitable, which may have influenced the study results. Third, influenza viruses accounted for more than two-thirds of non-SARS-CoV-2 associated ARDS, which limits the general interpretation of data on non-influenza respiratory viruses. Fourth, challenges in the management of critically ill patients with COVID-19 in our center make ventilator parameters difficult to record in medical charts and fully describe in our study. Fifth, data on SARS-CoV-2 associated ARDS were researched before Omicron was the dominant variant in South Korea; therefore, this limits the expansion of our data to Omicron-associated ARDS.

In conclusion, the present study showed several different aspects of the clinical characteristics of the two groups. Despite the lower severity of illness and organ dysfunction, patients with SARS-CoV-2 associated ARDS had similar but poorer clinical outcomes compared to those with non-SARS-CoV-2 respiratory viruses associated ARDS. 

## Figures and Tables

**Table 1:** Baseline characteristics of total patients, SARS-CoV-2, and non-SARS-CoV-2 -19 respiratory viruses.

Variables	Total	SARS-CoV-2	Non-SARS-CoV-2	*p*-Value
	*n* = 102	*n* = 31	*n* = 71	
Age, years old	69.5 (59.8–78.5)	67 (61–77)	70 (59–80)	0.578
Gender, male	64 (67.2)	14 (45.2)	50 (70.4)	0.025
BMI, (kg/m^2^)	24.1 (21.4–26.6)	25.5 (22.5–29.2)	23.2 (20.8–26.3)	0.015
CFS	3 (2–5)	2 (1–3)	3 (2–5)	<0.001
Hypertension	47 (46.1)	13 (41.9)	34 (47.9)	0.579
Diabetes mellitus	39 (38.2)	10 (32.3)	29 (40.8)	0.412
Chronic liver disease	10 (9.8)	3 (9.7)	7 (9.9)	1
Chronic heart failure	7 (6.9)	1 (3.2)	6 (8.5)	0.672
Chronic kidney disease	11 (10.8)	4 (12.9)	7 (9.9)	0.732
Cerebrovascular disease	18 (17.6)	2 (6.5)	16 (22.5)	0.05
Active malignancy	11 (8.6)	2 (6.5)	9 (12.7)	0.289
APACHE II	20 (15–26)	13 (11–18)	23 (19–27)	<0.001
SOFA	9 (5.8–11)	4 (4–6)	10 (9–13)	<0.001
Septic shock	42 (41.2)	0 (0)	42 (59.2)	0.001
Corticosteroid	58 (56.9)	31 (100)	27 (38)	<0.001
NM blocker	40 (39.1)	10 (32.3)	30 (42.3)	0.342
RRT	31(30.4)	6 (19.4)	25 (35.2)	0.109
HFNO before IMV	61 (59.8)	27 (87.1)	34 (47.9)	<0.001
Prone position	7 (6.9)	0 (0)	7 (9.9)	0.098
ECMO	23 (22.5)	9 (29)	14 (19.7)	0.301

BMI, body mass index; CFS, clinical frailty score; APACHE, acute physiology and chronic health evaluation; SOFA, sequential organ failure assessment; AKI, acute kidney injury; HFNO, high-flow nasal oxygen therapy; IMV, invasive mechanical ventilation; RRT, renal replacement therapy; ECMO, extracorporenal membrane oxygenation. NM means neuromuscular.

**Table 2:** Comparisons of laboratory, ventilator values, and microbiologic data at mechanical ventilation between SARS-CoV-2 and non-SARS-CoV-2 respiratory viruses.

Variables	Total	SARS-CoV-2	Non-SARS-CoV-2	*p*-Value
	*n* = 102	*n* = 31	*n* = 71	
WBC, ×10^3^/mm^3^	10.7 (4.4–16.5)	11.2 (8.7–16.8)	10.7 (3.4–15.6)	0.146
Hb, g/dL	12 (10.7–13.5)	12.2 (11.3–13.5)	11.9 (10.4–13.5)	0.390
Platelet, ×10^3^/mm^3^	175.5 (106.8–230)	199 (107–247)	172 (106–226)	0.480
Albumin, g/dL	2.9 (2.5–3.3)	3.1 (2.7–3.4)	2.8 (2.4–3.1)	0.007
Procalcitonin	0.9 (0.3–12.7)	0.3 (0.1–0.6)	3.63 (0.5–18.7)	<0.001
CRP, mg/dL	17.1 (12.5–28.5)	13.1 (7.7–16.1)	20.6 (13.3–32.5)	<0.001
D-dimer	3 (1.5–6.4)	2.1 (1.1–9.6)	3.3 (2.1–6)	0.381
NT-proBNP	1634 (363–8315)	525 (227–1256)	2516 (392–9434)	0.028
Lactate	2.4 (1.6–4.2)	1.8 (1.5–2.5)	2.7 (1.7–4.8)	0.011
PaO_2_:FiO_2_ ratio, mmHg	96.4 (73.8–137.1)	87 (71–110)	105 (76.7–142)	0.099
pH	7.34 (7.24–7.41)	7.38 (7.29–7.41)	7.33 (7.22–7.41)	0.088
PaCO_2_, mmHg	41 (33.7–47)	40 (34–47)	41 (33–47)	0.732
PEEP, cmH_2_O	10 (7.8–12)	10 (8–12)	8 (6–10)	0.007
PIP, cam H_2_O	27 (22–30)	28 (25–30)	25.5 (20–30)	0.108
Types of respiratory virus other than SARS-CoV-2				
Influenza			44 (62)	
Parainfluenza			7 (9.9)	
Respiratory syncytial virus			6 (8.5)	
Metapneumovirus			8 (11.3)	
Adenovirus			3 (4.2)	
Bocavirus			3 (4.2)	
Co-respiratory bacterial infection	39 (38.2)	3 (9.6)	36 (50.7)	<0.001
*S. pneumonia*		1 (3.2)	6 (8.5)	
*S. aureus*			17 (23.9)	
*K. pneumonia*			8 (11.3)	
*P. aerognosa*		1 (3.2)	0 (0)	
*Acinetobacter*		1 (3.2)	3 (4.2)	

WBC, white blood cell; Hb, hemoglobin; CRP, C-reactive protein; PaCO_2_, partial pressure of carbon dioxide; PEEP, positive end-expiratory pressure; PIP, peak inspiratory pressure; *S. pneumonia*, *Streptococcus pneumonia*; *S. aureus*, *Staphylococcus aureus*; *K. pneumonia*, *Klebsiella pneumonia*; *P. aeruginosa*, *Pseudomonas aeruginosa*.

**Table 3:** Clinical outcomes of total, patients with SARS-CoV-2, non-SARS-CoV-2 respiratory viruses.

Variables	Total	SARS-CoV-2	Non-SARS-CoV-2	*p*-Value
	*n* = 102	*n* = 31	*n* = 71	
Duration of MV, days	10 (4–20)	18 (11–36)	8 (4–14)	<0.001
Tracheostomy, *n* (%)	27 (26.5)	12 (38.7)	15 (21.1)	0.064
Ventilator liberation, *n* (%)	39 (38.2)	12 (38.7)	27 (38)	0.948
Length of hospital stay, days	18 (9–33.3)	28 (17–47)	14 (6–30)	0.001
In-hospital mortality, *n* (%)	55 (53.9)	18 (58.1)	37 (52.1)	0.579
28-day mortality, *n* (%)	42 (41.2)	10 (32.3)	32 (45.1)	0.227
60-day mortality, *n* (%)	55 (53.9)	17 (54.8)	38 (53.5)	0.902

MV, mechanical ventilation.

## Data Availability

Data are available upon reasonable request from the corresponding author.
